# Individual Differences in Cognition and Perception Predict Neural Processing of Speech in Noise for Audiometrically Normal Listeners

**DOI:** 10.1523/ENEURO.0381-24.2025

**Published:** 2025-04-22

**Authors:** Sana Shehabi, Daniel C. Comstock, Kelsey Mankel, Brett M. Bormann, Soukhin Das, Hilary Brodie, Doron Sagiv, Lee M. Miller

**Affiliations:** ^1^Center for Mind and Brain, University of California, Davis, Davis, California 95618; ^2^Institute for Intelligent Systems, University of Memphis, Memphis, Tennessee 38152; ^3^School of Communication Sciences & Disorders, University of Memphis, Memphis, Tennessee 38152; ^4^Neuroscience Graduate Group, University of California, Davis, Davis, California 95616; ^5^Psychology Graduate Group, University of California, Davis, Davis, California 95616; ^6^Departments of Otolaryngology | Head and Neck Surgery, University of California, Davis, Davis, California 95616; ^7^Neurobiology, Physiology and Behavior, University of California, Davis, Davis, California 95616

**Keywords:** auditory attention, auditory processing, cognition, event-related potential, N1 component, speech-in-noise

## Abstract

Individuals with normal hearing exhibit considerable variability in their capacity to understand speech in noisy environments. Previous research suggests the cause of this variance may be due to individual differences in cognition and auditory perception. To investigate the impact of cognitive and perceptual differences on speech comprehension, 25 adult human participants with normal hearing completed numerous cognitive and psychoacoustic tasks including the Flanker, Stroop, Trail Making, reading span, and temporal fine structure tests. They also completed a continuous multitalker spatial attention task while neural activity was recorded using electroencephalography. The auditory cortical N1 response was extracted as a measure of neural speech encoding during continuous speech listening using an engineered “chirped-speech” (Cheech) stimulus. We compared N1 component morphologies of target and masker speech stimuli to assess neural correlates of attentional gains while listening to concurrently played short story narratives. Performance on cognitive and psychoacoustic tasks was used to predict N1 component amplitude differences between attended and unattended speech using multiple regression. Results show inhibitory control and working memory abilities can predict N1 amplitude differences between the target and masker stories. Interestingly, none of the cognitive and psychoacoustic predictors correlated with behavioral speech-in-noise listening performance in the attention task, suggesting that neural measures may capture different aspects of cognitive and auditory processing compared with behavioral measures alone.

## Significance Statement

These findings contribute to our understanding of how cognition affects the neural encoding of auditory selective attention during speech perception. Specifically, our results highlight the complex interplay between cognitive abilities and neural encoding of speech in challenging listening environments with multiple speakers. By incorporating these additional measures of cognition, we can achieve a more comprehensive understanding of an individual's speech perception abilities, even in individuals with normal hearing. This approach could lead to earlier detection of hearing issues and more personalized interventions, ultimately enhancing communication outcomes for those with hearing difficulty.

## Introduction

Speech perception is a complex and multileveled process that requires the coordination of numerous neural systems, from decoding basic acoustic features of speech to transforming them into meaningful linguistic representations. Comprehension in noisy environments presents an additional challenge, placing substantial demands on every level of processing. This includes not only the encoding of stimuli and linguistic representations but also the segregation of auditory information into distinct streams (Griffiths and Warren, 2004), the ability to focus on a specific talker while filtering out others (Alain and Arnott, 2000; Shinn-Cunningham, 2008), and maintenance information over time (Martin, 2021). Consequently, speech-in-noise (SiN) performance is linked to both auditory and domain-general cognitive mechanisms (Akeroyd, 2008; Zekveld et al., 2013; Holder et al., 2018).

SiN perception disproportionately challenges listeners with hearing loss. However, many individuals with normal-hearing thresholds also struggle with speech comprehension in such conditions. In fact, as many as 15% of individuals seeking hearing assistance at audiology clinics have normal-hearing thresholds but identify difficulty understanding speech in noisy settings as their primary concern (Cooper and Gates, 1991; Hind et al., 2011; Tremblay et al., 2015). Even among listeners with normal thresholds, significant variability in SiN recognition can still be observed (Ruggles and Shinn-Cunningham, 2011).

This variability in SiN understanding could be influenced by individual differences in cognition and general perceptual abilities. Previous work suggests the most critical cognitive mechanisms for SiN performance include selective attention (Shinn-Cunningham, 2008), working memory (Gordon-Salant and Fitzgibbons, 1997; Akeroyd, 2008; Ronnberg et al., 2008), inhibitory control (Sommers and Danielson, 1999, Janse, 2012), and executive function (Perrone-Bertolotti et al., 2017). Deficiencies in these domains can significantly impair speech perception (Pichora-Fuller et al., 1995). In addition to cognition, nonaudiometric psychoacoustic abilities are also important, particularly sensitivity to temporal fine structure (TFS). TFS sensitivity is essential for spatial hearing, auditory object segregation and streaming, listening in the gaps, and pitch perception (Moore, 2008). As a result, TFS sensitivity is crucial for analyzing complex, ecologically valid auditory scenes, especially when speech is presented with background noise (Füllgrabe et al., 2015; Oberfeld and Klöckner-Nowotny, 2016). Notably, Oberfeld and Klöckner-Nowotny (2016) found that both cognitive measures, including selective attention, and psychoacoustic measures, such as TFS sensitivity, can predict SiN performance in individuals with normal hearing. Thus, explorations of SiN recognition abilities must also account for individual differences in both cognitive and basic auditory skills.

Additionally, successful SiN recognition relies on robust neural encoding mechanisms within the auditory system. The N1 component, part of the P1–N1–P2 complex, reflects cortical processing of sounds (Winkler et al., 2013). The N1 is considered an index of early perceptual encoding of speech sounds, and it is sensitive to acoustic cues within a stimulus, speech or otherwise (Getz and Toscano, 2021). N1 amplitude has been shown to reflect allocation of attentional resources to specific sounds, and it is sensitive to attentional modulation in multitalker listening scenarios (Picton and Hillyard, 1974; Stapells, 2009; Kerlin et al., 2010). Previous research suggests that N1 amplitude is larger for attended sounds compared with ignored ones, highlighting its role in auditory selective attention (Hillyard et al., 1973) and segregation of competing sound sources (Snyder et al., 2006; Gutschalk et al., 2007). Presumably, individual differences in speech perception should also be linked with variability of neural processes within the auditory cortex. Yet, despite the established influence of cognitive and nonaudiometric psychoacoustic abilities on speech perception, how these individual differences impact the neural encoding of speech (and SiN; Bednar and Lalor, 2020) remains unclear (for review, see Davidson and Souza, 2024). By investigating N1 amplitude differences for target and masker speech, our study aims to better characterize the variability in speech perception abilities among normal-hearing individuals in noisy environments.

The goal of this study is to understand how cognitive factors influence speech processing in the auditory cortex. We focus on N1 amplitude differences between target and masker speech to understand the brain's mechanisms for prioritizing relevant auditory information over irrelevant stimuli. We hypothesize that neural mechanisms involved in cocktail party listening are shaped not only by basic auditory factors, such as hearing sensitivity, but also by an individual's cognitive abilities. Specifically, we expect individuals who perform better on cognitive and psychoacoustic tasks to show larger N1 amplitude differences between target and masker speech. Enhanced cognitive and perceptual abilities are expected to improve auditory stream segregation, resulting in greater differentiation between the two speech streams. Collectively, these findings shed light on the cognitive contributions to neural mechanisms relevant for auditory selective attention during SiN perception.

## Materials and Methods

### Participant information

Twenty-nine participants were recruited for this study. Eligible participants were required to be between 18 and 40 years old, speak English as their first language, and report no neurological, psychiatric, or scalp conditions that could directly impair their ability to understand or attend to speech or hinder electrophysiological measurements (e.g., epilepsy, certain strokes, ADHD, scalp issues such as wounds). Participants were screened for visual acuity (<20/40 per Snellen chart), normal hearing (<25dB HL air-conduction thresholds between 250 and 8,000 Hz in both ears, no air–bone gaps >10 dB, no interaural asymmetries >20 dB at 500, 1,000, or 2,000 Hz), and cognitive abilities (>24 Montreal Cognitive Assessment score; Nasreddine et al., 2005).

One participant withdrew from the study due to personal reasons, two were excluded for failing the hearing screening at the time of testing, and one was excluded due to technical issues with data acquisition. Additionally, after fitting the model, one participant was identified as an influential outlier in the Trail Making Test (TMT) using a Cook's distance test threshold of 0.5 based on the multiple regression analysis (see below, Statistical analysis). This resulted in a final cohort of 24 participants. The average age of participants was 23.4 ± 5.02 SD years (range, 18–38 years). Fourteen participants were female and 10 were male. Twenty-one participants self-identified as right-handed, one as left-handed, and two as ambidextrous. On average, participants had 17.00 ± 3.18 SD years of education and 4.21 ± 3.82 SD years of formal music training. Pure-tone averages (PTA) were obtained for each ear as the average threshold at 0.5, 1, and 2 kHz. Average air-conduction PTA thresholds were 6.88 ± 3.78 SD and 7.04 ± 4.56 SD for the right and left ears, respectively. All participants provided informed consent as approved by the university's Institutional Review Board and were financially compensated for their participation and travel.

### Project overview

Each participant completed three sessions in total: a 1 h audiological exam, a 1.5 h behavioral session, and a 2.5 h laboratory electroencephalography (EEG) session. Most participants completed more than one session on the same day (e.g., audiological + behavioral or behavioral + EEG), with a long break in between to minimize fatigue. The analysis presented in this paper is based on a subset of data from a larger study investigating hearing across the lifespan, which will be detailed in future reports. Consequently, only a portion of the measurements and tests conducted during the study are included in this analysis.

### Audiology session

Participants completed a standard audiologic assessment either conducted by a professional audiologist or by highly trained personnel in the lab. Before inserting earphones, an otoscopic examination was conducted to check for tympanic membrane pathologies or any obstructions in the ear canal (i.e., cerumen) that could prohibit the use of insert earphones. Participants found to have ear canal occlusions or other visible pathologies were advised to consult their primary care provider and return to the study once the issue was resolved.

Pure-tone audiometry was performed for both ears either at a local affiliated audiology clinic or in a quiet, sound-dampened room within the lab. Air-conduction thresholds were measured using pulsed pure tones at frequencies of 0.25, 0.5, 1, 2, 3, 4, 6, and 8 kHz with insert earphones. Bone-conduction thresholds were measured using a RadioEar bone conductor placed on the mastoid at 0.25, 0.5, 1, 2, 3, and 4 kHz. The thresholds were determined using a 10 dB down/5 dB up staircase procedure. PTA were obtained for each ear as the average air-conduction threshold at 0.5, 1, and 2 kHz. While several other tasks typical of a standard audiology exam were conducted, only air- and bone-conduction measures are described here as they were the indicators used to determine normal hearing in participants.

### Cognitive tasks

Cognitive tasks were administered in a randomized order for each participant.

#### Flanker test

The Flanker test measures visuospatial interference, selective attention, and inhibitory control (Zelazo et al., 2013). We used a computerized version of the task using the PEBL software (Psychology Experiment Building Language; Mueller and Piper, 2014). In the task, five light gray arrows were displayed in the center of the screen against a black background. The arrows were either all pointing in the same direction (congruent condition) or with the center arrow pointing in the opposite direction to the flanking arrows (incongruent condition). Participants were asked to press the left or right shift keys on a keyboard corresponding to the direction of the center arrow. A fixation cross appeared for 500 ms before the arrows were presented, and participants had 2 s to respond before the trial timed out. A blank screen was shown for 2 s before the next trial began. Participants completed 8 practice trials, with 2 trials for each combination of conditions (left/right, incongruent/congruent), followed by 80 test trials (20 per condition). The interference effect was calculated as the median reaction time difference between incongruent and congruent trials.

#### Stroop

The Stroop test is a long-standing, well established assessment of linguistic interference and response inhibition (Stroop, 1935; Strauss et al., 2006). In our study, we utilized a computerized version of the Victoria Stroop Test (VST; Strauss et al., 2006), implemented using PEBL. The VST was selected for our study due to its shorter test administration time compared with other standardized Stroop tests. Evidence suggests that shorter test durations may be more sensitive to individual differences on this task (Klein et al., 1997). The VST has been proven effective with a wide range of adults, from 18 to 94 years old (Troyer et al., 2006).

As with other Stroop versions, the VST involves color identification of items presented to the participant, including a color word interference task. The VST is divided into three blocks always presented in the following order: colored dots (Part D), neutral words (Part W), and color words presented in contrasting text colors (Part C). Each block displays 24 items in a 6 × 4 grid on a gray background. Items are colored red, blue, green, or yellow, and participants press a corresponding number key to identify the color of each item. A square is shown around the current item trial, and it only moves to the next item with a correct response. The square flashes white when an incorrect response is made. Each color is used six times, and the item colors and the color-number keypad pairs are pseudorandomized for every participant (one of each color in each row). Part D serves as a practice block for color-number mapping and is not scored here. For Part W (neutral words) and Part C (color words), participants must name the colors in which the words are printed, disregarding the verbal content printed in lowercase letters (neutral words, when, hard, and, over; color words, red, green, blue, yellow). In Part C, the color name never corresponds to the text color, thus requiring individuals to inhibit an automatic reading response and only identify the text color itself. The interference effect is measured as the difference in median reaction times between Part C (i.e., the linguistic interference task) and Part W (i.e., the control task).

#### Reading span

Working memory ability was assessed using the reading span test (Daneman and Carpenter, 1980), as employed in previous research (Lunner, 2003; Rudner et al., 2012; Dryden et al., 2017). We administered a computerized version of the reading span via the PEBL program. The PEBL reading span test was developed for automated scoring as described by Unsworth et al. (2009). In this task, participants read sentences displayed on the computer screen and indicated whether each sentence made sense by pressing a key (i.e., in grammatical, coherent, or plausibility sense). Sentence lengths ranged between 9 and 14 words. After the sentence, a letter was presented on the screen for 1 s, and participants were required to memorize the sequence of letters presented across sentence–letter pairs. The task consisted of blocks of 3–7 sentence–letter pairs, with each block length presented three times in a random order throughout the test. At the end of each block, participants selected the correct sequence of letters from a 4 × 3 grid displayed on the screen and were instructed they could guess or leave a blank in the letter sequence if they were uncertain. They were then provided feedback regarding both sentence judgment and letter sequence accuracy.

Prior to the test, participants completed two practice examples each for sentence reading, letter memorization, and sentence–letter memorization pairs. The average reading time during these practice trials determined the timeout duration for the test blocks (i.e., timeout = 2.5 × average practice reading time; Unsworth et al., 2009). If participants did not respond to a sentence before the timeout period, the sentence trial was marked incorrect, and the subsequent letter was shown on the screen. Throughout the test, sentences, letters, and feedback were shown in white text on a black screen background.

Working memory span was determined by calculating the average accuracy of letter recall for three- and four-letter trials, when any letters were correctly recalled in the correct order (i.e., each correct letter contributing to the score). Though earlier research has suggested a working memory capacity of up to ∼7 items (Miller, 1956), recent studies indicate that this capacity is lower and can vary based on task demands and circumstances (Cowan, 2015). Consistent with findings indicating working memory capacity is ∼4 items (for reviews, see Cowan, 2001, 2015), our findings revealed that the accuracy for the three- and four-letter trials provided more stable and informative data for our model compared with the average accuracy across 3–7 letter trials (pilot data not shown). Therefore, we used the average accuracy from the three- and four-letter trials as our primary measure of working memory span.

#### TMT

The TMT (Reitan, 1958) was used to assess cognitive flexibility and executive function. In TMT-A, participants connect lines sequentially between numbers, while TMT-B requires them to alternate between numbers and letters (e.g., 1-A-2-B). Participants were given TMT-A followed by TMT-B, with a short practice at the beginning of each. The test was administered via PEBL, with white nodes displayed on a gray background and black text for letters and numbers. Participants clicked on the nodes in the correct sequence as quickly and accurately as possible, and lines were drawn on the screen to connect consecutive nodes upon correct selection. Given known limitations of the original Reitan-developed TMT-A/B (i.e., length differences between the nodes of the A and B versions; Gaudino et al., 1995), our version includes fixed node positions as mirrored images across TMT-A and TMT-B to ensure similar distance lengths for the two tests (Strelcyk et al., 2019). Any differences in reaction time are therefore presumed to reflect processing differences across the A and B versions. Executive function and cognitive flexibility were measured as the difference in median reaction times of TMT-B and TMT-A conditions.

#### TFS

The ability to localize low-frequency sounds along the horizontal plane relies on interaural time differences (ITD), which are important cues for distinguishing multiple sounds such as recognizing speech from a target talker amidst spatially separated background noise or other interfering sounds (Moore, 2021). For sinusoidal signals, ITDs are equivalent to interaural phase differences (IPDs), and the ability to discriminate changes in IPDs depends on binaural sensitivity to TFS. To assess IPD discrimination abilities, we used the TFS-LF (low-frequency) test (Hopkins and Moore, 2010), which is implemented in a computerized software program developed by Sęk and Moore (2012, 2021). In the TFS-LF test, participants are tasked with discriminating sinusoidal tone bursts with adaptively varying IPDs, where the tone envelopes are synchronized across both ears, ensuring that task performance relies on sensitivity to TFS (IPD). TFS-LF administration was consistent with previous work and is briefly described below (Hopkins and Moore, 2010; Sęk and Moore, 2012, 2021; Füllgrabe and Moore, 2018).

IPD discrimination was evaluated at test frequencies of 250 and 500 Hz, consistent with previous studies (for review, see Füllgrabe and Moore, 2018). The intensity level was set individually for each ear 30 dB above the participant's pure-tone thresholds at the tested frequencies (i.e., 30 dB sensation level at 250 and 500 Hz). The TFS-LF task consisted of a two-interval, two-alternative forced–choice design, where each interval included four successive tones. In one of the intervals, all four tones had a consistent 0° IPD, while in the other interval, the IPD alternated between 0° and ϕ across the four successive tones. A 0° IPD is perceived as a tone localized to the center of the head, whereas a large IPD is perceived as a tone lateralized (or as more diffuse) toward one ear. Participants were asked to identify the interval in which the tones sounded different or appeared to change in some way. Each tone had a duration of 400 ms, with 20 ms rise and decay times and a 300 ms interinterval gap between tone blocks. Feedback was provided after each trial. The IPD ϕ was adaptively varied, starting from 180° (maximally lateralized), using a two-down, one-up procedure to estimate a threshold corresponding to 71% correct responses. That is, the value of ϕ is divided by a factor *k* after two successive correct responses or multiplied by factor *k* following one incorrect response. Before the first turn point, *k* = 1.253; between the first and second turn points, *k* = 1.252; and *k* = 1.25 after the second turn point. The threshold was computed as the geometric mean value of ϕ at the last six turn points, and the final TFS-LF measure was then calculated as the average across both 250 and 500 Hz frequencies.

#### Additional tests

During the cognitive session, participants also completed two additional tests: (1) a pitch discrimination task and (2) a speech recognition in noise test, which assesses SiN perception. These measures were not included as predictors in the current analysis, as our primary focus was on evaluating variables that most closely align with existing research on predictors of SiN perception (Akeroyd, 2008; Shinn-Cunningham, 2008; Janse, 2012; Perrone-Bertolotti et al., 2017).

### Continuous multitalker spatial attention task

To evaluate SiN perception abilities, participants completed a continuous multitalker spatial attention task.

#### Stimuli

The stimuli consisted of short fairytale stories available in the public domain (available for listening on www.librivox.com). Stories were selected to have content that was unlikely to be widely recognized, reducing the chances of participants relying on modern renditions for comprehension recall (cf. stories popularized by children's books or movies). Additionally, each story was chosen to have a similar narrative arc—i.e., up through rising action, climax, or early falling action—within a 7.5 min block to ensure sufficient and consistent content for probing comprehension. Thirteen stories were piloted with four raters who evaluated them based on factors such as interest and engagement, ease of understanding, complexity of grammar or writing style, familiarity (i.e., low scores preferred), and suitability for an adult audience (not only for children). The seven highest-scoring stories across these criteria were selected for use in the experiment.

The short stories were revised to incorporate monosyllabic color words as targets for the experiment (e.g., red, green, black, white, etc.). The color words were integrated into the story itself rather than being inserted as unrelated, random target words (e.g., “…with a narrow *blue* ribbon over her shoulders”). A male native English speaker with an American Midwestern accent recorded the modified stories using a Shure KSM244 vocal microphone (cardioid polar pattern, high-pass filter at 18 dB per octave with an 80 Hz cutoff) and Adobe Audition [sample rate, 48,000 Hz; 32 bit depth (float)] in a soundproof, quiet room. Silent gaps in the recordings longer than 500 ms were shortened to 500 ms, following procedures similar to those in previous studies (Broderick et al., 2018; Teoh and Lalor, 2019; Teoh et al., 2022). Finally, the edited recordings were cropped to 7.5 min per story.

#### Cheech

The short story auditory stimuli were subsequently modified using a patented technique known as “Cheech” (“chirped-speech”), which is intended to elicit robust auditory evoked potentials from the brainstem to the cortex (Backer et al., 2019; Miller et al., 2020). In addition to providing insight into multiple levels of neural processing simultaneously (e.g., ABR, MLR, LLR), Cheech provides greater acoustic control over the specific acoustic parameters in naturalistic speech used to evoke the brain responses. We describe the details in this paper for completeness, but some of the details may not be relevant for LLRs. In Cheech, some of the glottal pulse energy within specified frequency bands is replaced with narrowband synthetic chirps (for more details, see Backer et al., 2019). These chirps are aligned with the natural glottal pulse energy in voiced speech, creating an acoustically fused auditory perceptual object. This modification maintains the naturalistic qualities and linguistic content of speech while introducing sufficient transient chirp activation to measure auditory brainstem, thalamic, and cortical responses.

The process begins by identifying voiced epochs where chirps will be inserted. Audio was filtered from 20 to 1,000 Hz, and voiced periods of at least 50 ms were defined when the speech envelope between 20 and 40 Hz exceeded a threshold of ∼28% of the overall speech root-mean-square (RMS) amplitude. The timing of the glottal pulses within these voiced periods was determined through a speech resynthesis process using a custom MATLAB code (The MathWorks Inc., 2021) and the TANDEM-STRAIGHT toolbox, which retains the original speech periodicity characteristics (Kawahara et al., 1999, 2008; Kawahara and Morise, 2011). The continuous speech was then refiltered into alternating, octave-wide frequency bands, 0–250, 500–1,000, 2,000–4,000, and 11,000–∞ Hz, with chirp energy constrained to frequency bands of 250–500, 1,000–2,000, and 4,000–11,000 Hz. The chirp and speech bands in the alternating, interleaved octave-wide bands were then added together. Each mono track was then duplicated to form stereo audio. Before the Cheech synthesis, audio files were normalized to ensure consistent chirp amplitudes relative to the speech levels across each story, and the RMS amplitudes were rechecked across all Cheech-modified stories to ensure equivalence after the Cheech process was completed.

The timing of the chirps varied with the natural fluctuations in the running speech, with special modifications to optimize measurement of auditory event-related potentials. Specifically, when voicing energy was detected, chirps were inserted at minimum 18.2 ms apart (55 Hz), skipping glottal pulses that occur within the 18.2 ms window. The first chirp in any sequence was always followed by a minimum interval of 48 ms before the next chirp was presented, skipping glottal pulses in between. This longer ISI was designed to enhance the measurement of middle-latency responses (MLRs); we use this “first chirp” to elicit LLR N1s as well.

In the dual-talker conditions, one story from each pair was pitch and vocal tract modulated using MATLAB STRAIGHT prior to the Cheech process. Specifically, stories intended to represent a female voice were pitch modulated to an average fundamental frequency (F0) of 180 Hz, while the original male voice audio had an average F0 of ∼128 Hz. By modulating the F0, key speaker characteristics such as phrasing, intonation, and pacing were maintained, as all stories were initially recorded by a single talker. Additionally, this pitch modification created a perceptual distinction between the two voices, introducing a combined voice gender and spatial release from masking effect. This effect, which is consistent with prior findings (Oh et al., 2021, 2022), facilitated better differentiation of the spatially separated voices and improved task performance during pilot testing.

#### Continuous multitalker spatial attention task

**Figure 1.**
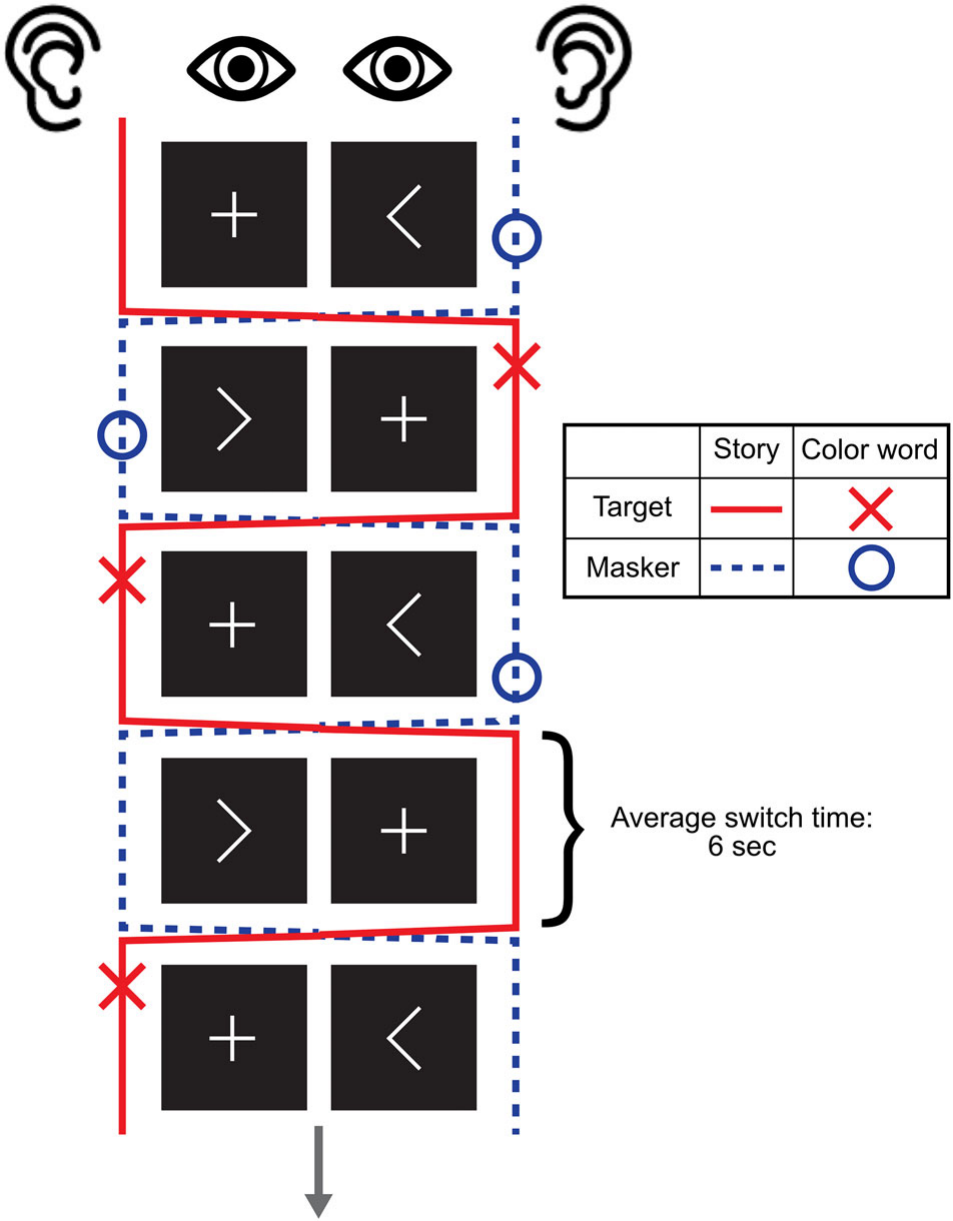
Continuous multitalker spatial attention task procedure. The figure illustrates the visual cues presented on screen to participants during the task while they listened to a series of short stories. The target and masker (when present) alternate between spatial locations. Only the target story is present in the mono-talker condition, but the spatial location of the talker still switches sides as shown by the red line. Participants are instructed to focus on the side of the screen indicated by the “+” symbol, which marks the location of the target story. They are also instructed to press the spacebar when they hear a color word spoken by the target speaker (red “X” symbols) and ignore any color words in the masker story (blue “O” symbols).

The Cheech stimuli were spatially filtered using head-related transfer functions (HRTFs) from the SADIE II database (Armstrong et al., 2018) to simulate audio at ±15°. This spatialized audio was mirrored by two symbols on two computer screens centered ∼15° to the left and right of the participant's midline. The symbols “<” and “>” indicated the target story's location on the left or right, respectively, while a “+” symbol denoted the location of the target story, as shown in Figure 1. The visual symbols were identical in size, color, and line lengths to ensure consistent luminance for eye-tracking measurements (not analyzed in this report). The custom MATLAB Psychtoolbox code (Brainard, 1997; Pelli, 1997) was used to program the spatial and visual stimuli to switch between left and right locations 75 times over a 7.5 min period, with each switch occurring approximately every 6 s (random distribution of 6 ± 1 s). The visual icons switched instantaneously at each switch cue, while the audio crossover fade time was set to 35 ms, starting at the onset of each switch cue. During piloting, this fade duration was found to effectively minimize audio artifacts, such as “pops,” while also reducing the perception of a gradual audio glide between spatial locations (i.e., as sudden of a jump as possible without introducing noticeable distortions).

Seventy-five switches were placed in the story, occurring every 6 s on average. Twenty-five of those switches were specifically timed to precede the color words. The time intervals between these switches and the color words were 0.125, 0.25, 0.5, 1, and 2 s, with the intervals randomly assigned to the color words so that each interval duration occurred exactly five times. These intervals were determined based on preliminary piloting that aimed to evaluate whether brief lapses in attention following a shift in spatial focus—analogous to “attentional blinks”—affected participants’ ability to detect color words; these results will be presented in a subsequent paper. Additionally, the 25 color words were evenly distributed between left-to-right and right-to-left switches within each story, minus the one remainder. Onset timings for the color words were calculated using Gentle, an audio forced aligner (https://github.com/lowerquality/gentle). The Gentle forced aligner software finds the start and end points of spoken words, allowing us to pinpoint exactly when the color words start, which was needed for determining switch times.

#### Experimental conditions

After cropping the audio, the seven stories were assigned to specific experimental conditions: two stories presented alone (“mono-talker”), two sets of paired stories (“dual-talker”), and one for initial practice. Half of the stories (one mono-talker and one dual-talker pair) had modified Cheech characteristics to study their effects on brainstem encoding (results forthcoming in future reports); however, this modification did not impact cortical responses, so we used all six nonpractice stories for our N1 analyses. Since our focus is on predicting SiN performance, this paper primarily examines the dual-talker conditions (excluding Fig. 4 and associated analyses). In the dual-talker paradigm, each story was presented twice—once as the “target story” and once as the “masker story” within its pair. This experimental manipulation allowed us to assess the effects of directed spatial attention, as participants were instructed to either “attend” to or “ignore” the same audio depending on its role in the trial. The order of stories and conditions was counterbalanced across participants using a Latin square design to minimize potential sequence-dependent learning effects. Additionally, no pair of stories was presented consecutively.

Story pairs in the dual-talker condition required additional considerations. As described above, Cheech, one voice in each story pair was pitch and vocal tract modulated to simulate a female speaker. Consistent with findings on the combined effects of voice gender and spatial release from masking (Oh et al., 2021, 2022), two distinct talker F0s facilitated better listener differentiation between spatially separated voices and improved task performance during piloting. The mono-talker stories were presented in their original, unmodified male voice. To minimize the influence of stimulus variability on the N1, the analysis in this paper focused solely on the male target and male masker conditions. Due to the naturalistic embedding of color words within the stories, some overlap occurred in the dual-talker pairings, i.e., color words in the target and masker speech occurred in close temporal proximity. Story pairs were selected to minimize color word overlaps, resulting in six overlapping words that were excluded from the behavioral analysis (four from one story pair and two from the other pair). A few target story switch times were adjusted to ensure that no color words in the masker story occurred during the switch (i.e., masker color word onset was not between −0.5 and 0.1 ms from a switch time).

#### Stimulus presentation equipment

Participants were seated in a soundproof, electromagnetically shielded booth ∼176 cm from two computer monitors. The subjects and cables were positioned at a sufficient distance from the monitors. Testing confirmed that this arrangement effectively prevented any electromagnetic interference from the monitors. The Dell UltraSharp monitors had a 60 Hz refresh rate with screen settings maintained at standard factory defaults. Stimulus presentation, including both audio and visual components, was controlled using a custom MATLAB and Psychtoolbox code. Auditory stimuli were routed from a Hammerfall audio card to an RME Fireface UFX II audio interface, then through an RME ADI-2DAC FS headphone amplifier, and finally delivered via ER-2 insert earphones (Etymotic Research/Interacoustics). The ER-2 insert wires were electromagnetically shielded up to the transducer box to minimize EEG artifacts (Campbell et al., 2012). The Cheech stimuli were presented at 75 dB SPL.

In addition to the two-channel audio tracks, a pair of channel outputs carrying trigger events was routed from the Fireface audio interface to two Brain Products StimTraks. These StimTraks monitored triggers for the target and masker stories, respectively, converting the audio trigger signals into pulses sent to a TriggerBox (Brain Products). This configuration ensured that audio playback was synchronized with the chirp triggers embedded during the Cheech process, minimizing latency jitter, and maintaining sample-level synchronicity. Triggers managed by Psychtoolbox—such as participant responses, block sequence triggers, and spatial location switches—were also routed to the TriggerBox through the computer parallel port. The TriggerBox then transmitted both parallel port and StimTrak triggers to the BioSemi signal receiver box (refer below, EEG recordings). To calibrate our stimuli, we used a Larson-Davis PRM902 preamplifier connected to a Larson-Davis 2221 preamplifier power supply, paired with a B&K Ear Simulator Type 4157 (IEC 60318-4 Coupler). Audio stimuli were calibrated using either C-weighting (for Cheech stimuli) or *Z*-weighting (for cognitive and SiN tests), with a default preamplifier gain of 20 dB applied. Signal intensity measurements were then analyzed using a custom MATLAB script to ensure accuracy and consistency across all stimuli.

#### Attention task procedure

The order of events within each block is as follows: a 1 min rest period (for resting state EEG analysis), 7.5 min of audio presentation, comprehension questions, subjective workload questions, a flash reaction time task, and a break period. During the rest period, participants were instructed to remain still with their eyes open. They were instructed to attend to the target story as the auditory stream alternates between left and right spatial locations. They must immediately shift both their selective attention and eye gaze to the fixation point (“+”) on the computer screen as it switches the cue. Participants were also instructed to press the spacebar upon hearing the embedded color words in the story as quickly and accurately as possible. In dual-talker condition blocks, they were instructed to ignore color words from the masker story and only respond to those from the target story. Following the audio presentation, participants completed various behavioral tasks, including comprehension questions, subjective task workload assessments, and reaction time tests which are not discussed in this paper. Finally, participants were given a break before starting the next block. The entire experiment presentation was controlled by a custom MATLAB and Psychtoolbox code.

#### Measure of SiN perception

SiN perception during listening was measured by the correct identification of color words embedded in the target story, referred to as “hits.” A hit was defined as pressing a designated keyboard button within 2 s after the onset of a target word. Any color words in the dual-talker condition that occurred within ±2 s of each other in the target and masker were excluded from analyses. Specifically, the relevant measure was the proportion of correct hits out of the total possible, nonoverlapping target words (*n* = 25 in the mono-talker condition, *n* = 21 or *n *= 23 in the dual-talker condition, depending on the story pair).

### EEG

#### EEG recordings

EEG recordings were obtained in an electrically shielded booth using a BioSemi ActiveTwo system (BioSemi, www.biosemi.com) with a sample rate of 8,192 Hz from 64 electrodes following the International 10–20 standard, plus an additional 4 electrode pairs around ears (earlobe, mastoid, posterior to the earlobe and inferior to the mastoids, and superior–anterior to the tragus near the zygomatic arch) for 72 total electrodes. Prior to recording, offsets from all electrodes were measured to be below 20 µV relative to the common mode sense electrode using the ActiveView2 software. Data were recorded using the Lab Streaming Layer (LSL) software (https://github.com/sccn/labstreaminglayer) in XDF format on a desktop computer running Windows 10. Events were recorded alongside EEG data as a separate trigger channel in the XDF file.

#### EEG preprocessing

The EEG data were preprocessed in MATLAB 2021b using EEGLAB (Delorme and Makeig, 2004) and a custom MATLAB code. Data were first imported from XDF files into EEGLAB using the XDF importer plugin (version 1.19, https://github.com/xdf-modules/xdf-EEGLAB) after which events were extracted from the trigger channel using a custom MATLAB code. Given the high number of densely packed event codes (>130,000/subject at an average rate of one event every 20 ms), events from the three different event streams (parallel port and two StimTraks) often overlapped in the trigger channel, causing occasional event code errors. We therefore checked all recorded events against expected events and corrected for any missing, extra, or jittered events using a custom MATLAB code. We then applied a second set of corrections to adjust for differences in the acoustic signal and the event times. These corrections accounted for the 1.973 ms audio delay due to the HRTF process, a 1 ms audio delay due to the audio travel time from the transducer via the insert earphone tubes, a 1 ms trigger delay in one of the StimTrak channels caused by the TriggerBox hardware, and a 0.541 ms trigger delay to the chirp triggers accounting for a difference between the expected chirp timing from the Cheech process and the chirp onsets.

Data were then pruned to retain only sections of the data from the rest periods and the attention task periods with 10 s buffers on both sides of the selected periods to prevent filtering artifacts. Each remaining section was then DC corrected and filtered using a noncausal second-order 0.1 Hz high-pass Butterworth filter using ERPLAB's filter function to remove slow drifts and then referenced to electrode Cz. Data were then checked and cleaned for 60 Hz line noise, which was occasionally found in some channels despite the electrically shielded booth. Only channels with detected line noise were cleaned (mean, 4.5 channels ± 7.7 SD; range for a given subject, 0–39 channels) to minimize the effects of the cleaning. This was achieved by first checking for line noise using a frequency tagging approach. Individual channel spectra were calculated across all frequencies. For each frequency, *x*, the average spectral power of four neighboring frequencies except those immediately adjacent (*x* − 2, *x* − 3, *x* + 2, *x* + 3) were subtracted. If the resulting neighbor corrected power at 60 Hz or any of its first four harmonics (120, 180, 240, 300 Hz) was greater than eight standard deviations calculated from the corrected power from all other frequencies, that channel was cleaned of line noise using the Cleanline plugin (https://www.nitrc.org/projects/cleanline). This repeated up to a maximum of five times or until no further line noise was detected.

We used independent component analysis (ICA) to further clean the data of eyeblink, eye movement, and heartbeat artifacts following the methodology outlined by Luck (2022). This involved making a copy of the data processed up to this point (ICA copy), downsampling, and cleaning the ICA copy, followed by ICA and marking and rejecting artifact components and then transferring the cleaned ICA weight matrix from the ICA copy to the original data. This approach allows for a massive reduction in ICA computation time while still retaining all the information in the data relevant for decomposing eye and heart artifacts. Specifically, after making a copy for ICA, we downsampled to 256 Hz to speed ICA processing and then manually inspected and rejected bad channels (mean, 3.67 ± 2.16 SD channels; range = 0–9 channels). Following channel rejection, a noncausal 1–50 Hz eighth-order Butterworth bandpass filter using ERPLAB's filter function was applied. We then removed sections of data with significant movement and muscle artifacts that can hinder ICA's ability to isolate eye and heart components, using ERPLAB's continuous artifact rejection function (mean, 126.54 ± 86.85 s SD or 3.52 ± 2.36%; range, 4.98–298.79 s or 0.14–7.96%). The rejection process operated with a 1 s sliding window with a 0.25 s step size and an initial rejection threshold of ±200 µV. In order to retain eye activity for ICA, the rejection threshold was applied against all channels except those mostly likely to contain eye-related activations (FP1, FPz, FP2, AF7, AF3, AFz, AF4, AF8). The rejection threshold was inspected and adjusted for each subject to maximize rejecting movement and muscle artifacts while retaining eyeblinks (mean, 220.83 ± 29.18 SD µV; range = 200–300 µV). We then used the Infomax ICA algorithm after first using principal component analysis to reduce the number of channels from an average of 67–32 for each subject to further speed ICA. ICA components constituting eyeblinks, eye movements, and heart artifacts (when present) were manually selected for rejection (mean, 2.79 ± 0.66 SD components; range, 2–4 components). The resulting ICA weight matrix was then transferred to the original dataset, after first rejecting the same bad channels in the original dataset as were identified in the ICA dataset.

After the data were cleaned with ICA, we downsampled it to 512 Hz and applied a noncausal 0.5–40 Hz eighth-order Butterworth bandpass filter using ERPLAB's filter function. Data were then re-referenced to the average of the left and right earlobes. In the case that one or both earlobe channels were rejected as bad, the average of the left and right mastoid electrodes was used as reference instead. While re-referencing, activity at channel Cz (the previous reference) was recalculated and placed back into the data. We then used spherical interpolation on any channels rejected.

EEG data were epoched −50 to +500 ms from the first chirp of each word in each condition, with the prestimulus period used as the baseline. Each condition had roughly the same number of events (MonoMale, 2,417; DualTargetMale, 2,392; DualMaskerMale, 2,392), except for one subject in which data recording ended early due to computer error (MonoMale, 1,249; DualTargetMale, 2,000; DualMaskerMale, 2,392). Epochs with artifacts were cleaned with a two-step process: first, a voltage threshold of ±300 µV was applied to remove any epochs with extreme artifacts. Second, any remaining epochs with voltage activity outside of 6 SD for a single channel or 2 SD across all channels were rejected (MonoMale, mean, 5.01 ± 0.83% SD rejected epochs; range, 3.07–6.85%; DualTargetMale, mean, 4.84 ± 0.86% SD rejected epochs; range, 3.75–7.02%; DualMaskerMale, mean, 5.12 ± 1.1% SD rejected epochs; range, 2.9–8.12%).

#### N1 extraction

N1 component activity was captured as the mean amplitude at electrode Fz between 110 and 160 ms postchirp onset. The electrode site Fz was chosen based on previous research using the Cheech paradigm (Backer et al., 2019) and literature on the scalp regions where auditory evoked responses generally reach peak amplitudes (Kappenman and Luck, 2011). Given the N1 component lacked a clear peak (as is typical during continuous speech presentation) and was usually followed by a sustained negativity (Figs. 3, 4), a mean amplitude over the 50 ms interval was used instead of more traditional peak fitting approaches, with the time course chosen based on visual inspection of mono and dual target conditions (Fig. 4). Difference scores were calculated by subtracting mean dual target N1 amplitudes from mean dual masker N1 amplitudes.

### Statistical analysis

All statistical analyses were performed using R version 2023.09.1 + 494 (Posit Software, PBC) on macOS Sonoma 14.6.1. A paired *t* test was performed to evaluate the statistical significance of differences between target and masker N1 amplitudes. A linear multiple regression analysis was conducted to examine how N1 amplitude differences between attended (target) and unattended (masker) speech relates to cognitive and nonaudiometric psychoacoustic predictors. The criterion variable was the N1 amplitude difference in the continuous multitalker spatial attention task. The five predictors included (1) median reaction time differences between the incongruent and congruent conditions of the Flanker task, (2) median reaction time differences between the incongruent and congruent conditions of the Stroop task, (3) median reaction time differences between TMT-B and TMT-A, (4) letter recall accuracy from three- and four-letter trials of the Reading Span test, and (5) average threshold of the TFS task across 250 and 500 Hz conditions.

The five predictors were entered simultaneously into the multiple regression model to assess their collective effect on the N1 amplitude differences. Prior to the analysis, several diagnostic checks confirmed the suitability of multiple regressions. Residual versus fitted plots and *Q*–*Q* plots indicated no significant deviations from normality. Multicollinearity among predictors was assessed using variance inflation factors (VIFs), with values above 5 suggesting problematic multicollinearity (Marcoulides and Raykov, 2019). The highest VIF in our model was 1.43, indicating no substantial effects of multicollinearity. Influential outliers were evaluated using a Cook's distance threshold of 0.5. As mentioned previously, one influential outlier was detected and subsequently removed, resulting in a final sample size of *N* = 24. Plots of each predictor against N1 amplitude differences demonstrated no significant deviations from linearity. With a sample size of 24 and five predictors, the subject-per-variable ratio (SPV) was 4.8. A linear regression model requires a minimum SPV of 2 for reliable estimation of regression coefficients, standard errors, and confidence intervals (Austin and Steyerberg, 2015). All variables were *z*-standardized prior to regression analysis.

Following the multiple regression analyses, dominance analysis was performed to assess the relative importance of predictors in explaining the variance in N1 amplitude differences (Budescu, 1993). This method evaluates the dominance of each predictor by comparing their *R*^2^ contributions across all possible subset models. Specifically, each predictor is added to all possible models that do not already include it, and the increase in *R*^2^ is measured. This additional analysis was necessary because the regression coefficients can be influenced by even moderate collinearity among predictors, potentially skewing the assessment of each predictor's contribution (Mizumoto, 2023). A predictor's general dominance weight (GDW; Azen and Budescu, 2003) is a measure of the proportion of the variance explained by each predictor. It is calculated by averaging the squared semipartial correlations for the predictor across all possible subset models. Higher GDW values indicate greater explanatory power of a predictor when combined with other predictors. The total sum of the GDWs equals the proportion of variance explained by the regression model, *R*^2^. Similar analysis approaches have been used in previous studies, such as Oberfeld and Klöckner (2016) and Oberfeld et al. (2024).

To validate the predictive accuracy of the regression model, we employed leave-one-out cross–validation (LOOCV). This technique was utilized to ensure the model's robustness in predicting N1 amplitude differences based on participants’ cognitive and psychophysical performance. In LOOCV, one observation in the dataset (one participant) is used as a validation set, while the remaining datasets (N-1) are used as training data to predict the value for the omitted observation. This iterative process is repeated *N* times, once for each observation. For each iteration, the model's prediction error is calculated, and these errors are aggregated to compute the RMS error (RMSE), which provides a measure of the average magnitude of prediction errors. This method provides a nearly unbiased estimate of the model's predictive performance, particularly beneficial for smaller datasets.

A separate multiple regression analysis was conducted using the same five predictors to examine their ability to predict the proportion of color word hits in the continuous multitalker spatial attention task. The predictors were entered simultaneously into the regression model to assess their combined impact on speech perception performance in noise. Prior to the analysis, the appropriateness of using multiple regression was verified through several diagnostic checks. Although the residuals versus fitted plots and *Q*–*Q* plots indicated some deviations from normality, no transformation of the response variable significantly improved the distribution of the residuals or altered the results. Since the same predictors were used, the multicollinearity checks from the first model remained unchanged. The influential outlier observed in the first model was also removed in this analysis (*N* = 24). Plots of each predictor against N1 amplitude differences showed no significant deviations from linearity. All variables were *z*-standardized prior to regression analysis.

## Results

### Cognitive and psychoacoustic predictors

**Figure 2.**
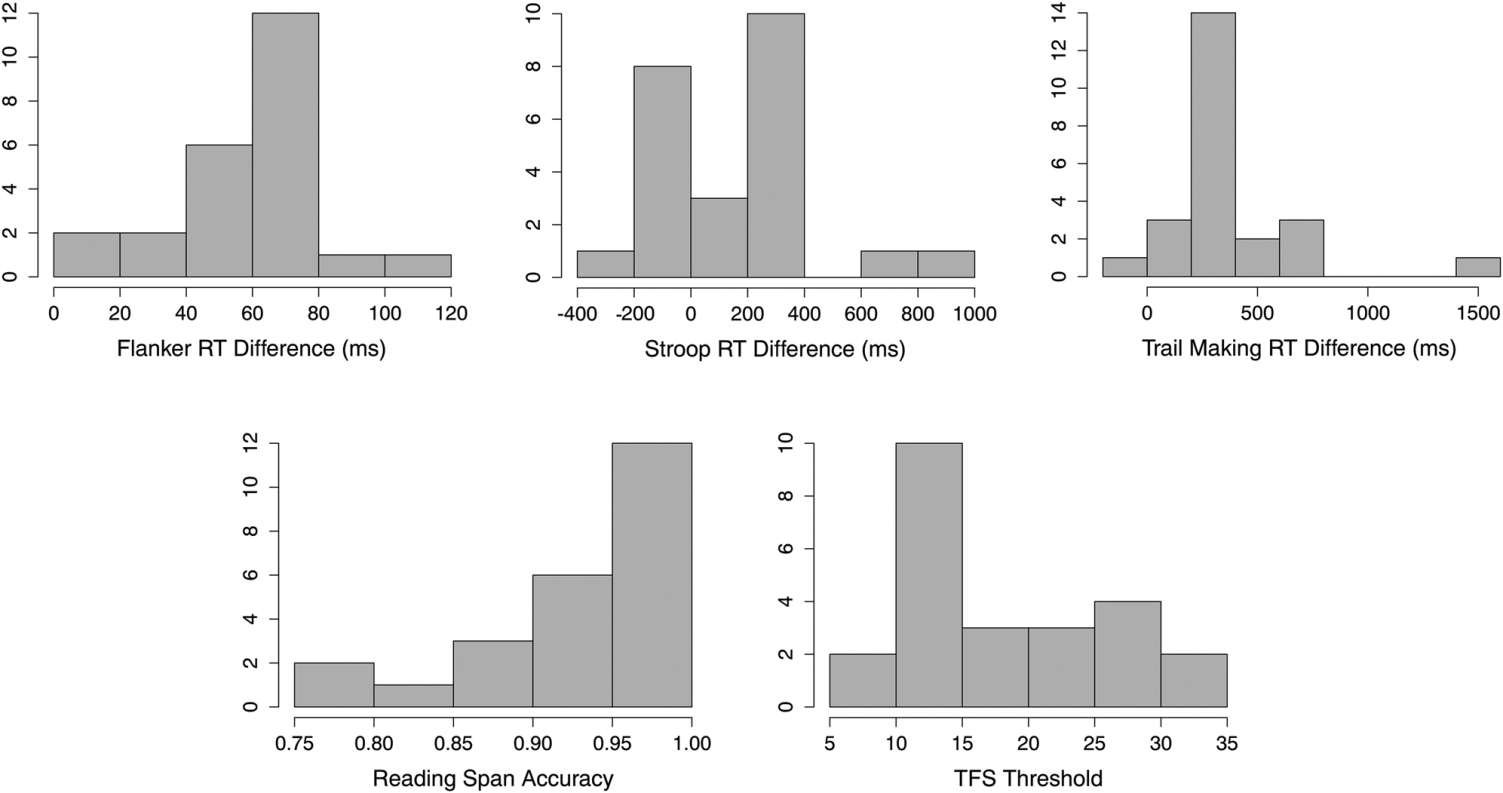
Histograms illustrating the distribution of performance scores across five cognitive and psychoacoustic predictors. Larger RT difference scores (Flanker, Stroop, and Trail Making) indicate greater response (cognitive) interference in each domain (i.e., slower RTs for the incongruent/alternating trials than the congruent/sequential trials).

Figure 2 presents box plots illustrating the distribution of scores across the five cognitive and psychoacoustic tasks administered to participants, highlighting the variability in performance. While some plots reveal visually apparent outliers, these data points did not influence the predictive performance of the models and were therefore retained in the analysis.

### N1 across conditions

**Figure 3.**
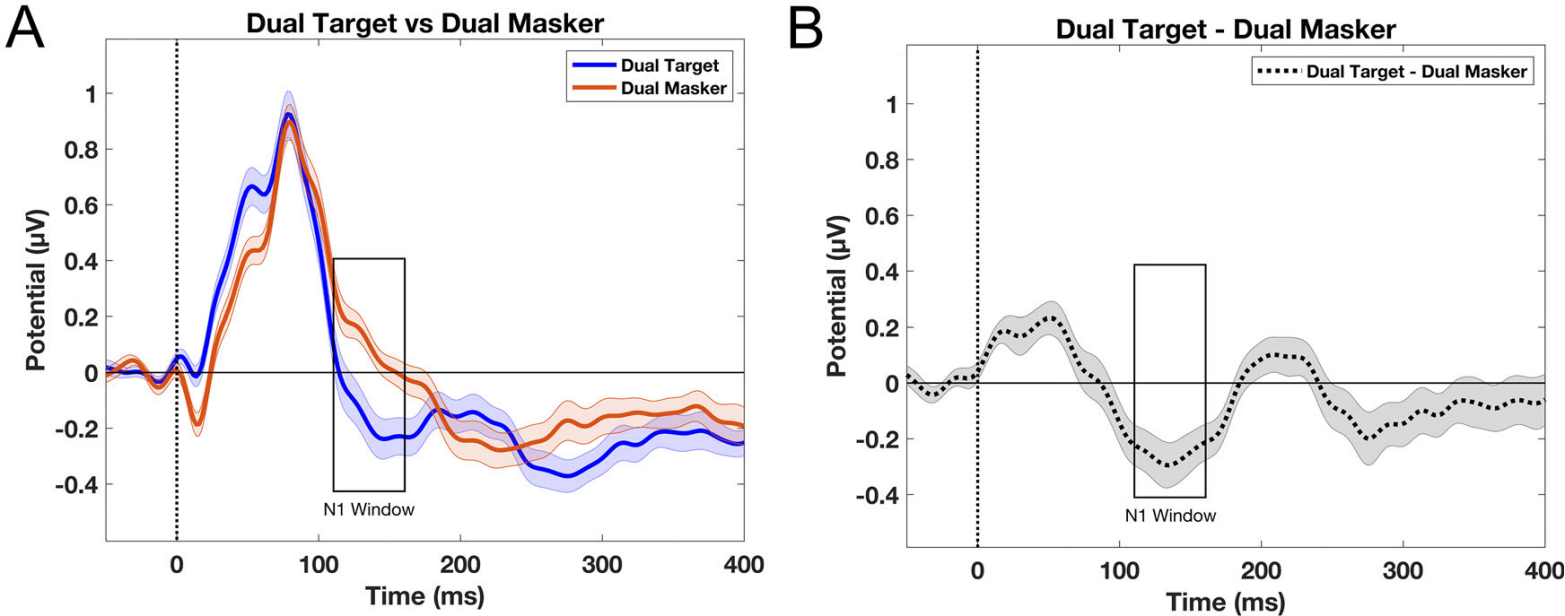
Attentional modulation of speech-evoked neural activity in dual-talker conditions at electrode Fz. Error bars indicate standard error. ***A***, Grand average ERP waveforms of the target and masker speech. ***B***, Difference waveforms between target and masker stories.

The grand average waveforms in Figure 3*A* reveal a significant difference in N1 amplitudes between the target and masker story conditions. The N1 amplitude was significantly larger for the target story condition (M, −0.142 µV; SD, 0.350 µV) compared with the masker story condition (M, 0.113 µV; SD, 0.198 µV). A paired *t* test confirmed this difference as statistically significant (*t*_(23)_ = −3.664; *p* = 0.001). Figure 3*B* displays the difference wave, created by subtracting the ERP waveform of the masker from that of the target. Negative N1 amplitude differences indicate a stronger N1 response to target speech compared with masker speech, while positive differences suggest a stronger response to the masker than to the target. The average N1 amplitude difference between the target and masker speech was −0.254 ± 0.340 µV, indicating a stronger (more negative) N1 response to the target speech compared with the masker.

**Figure 4.**
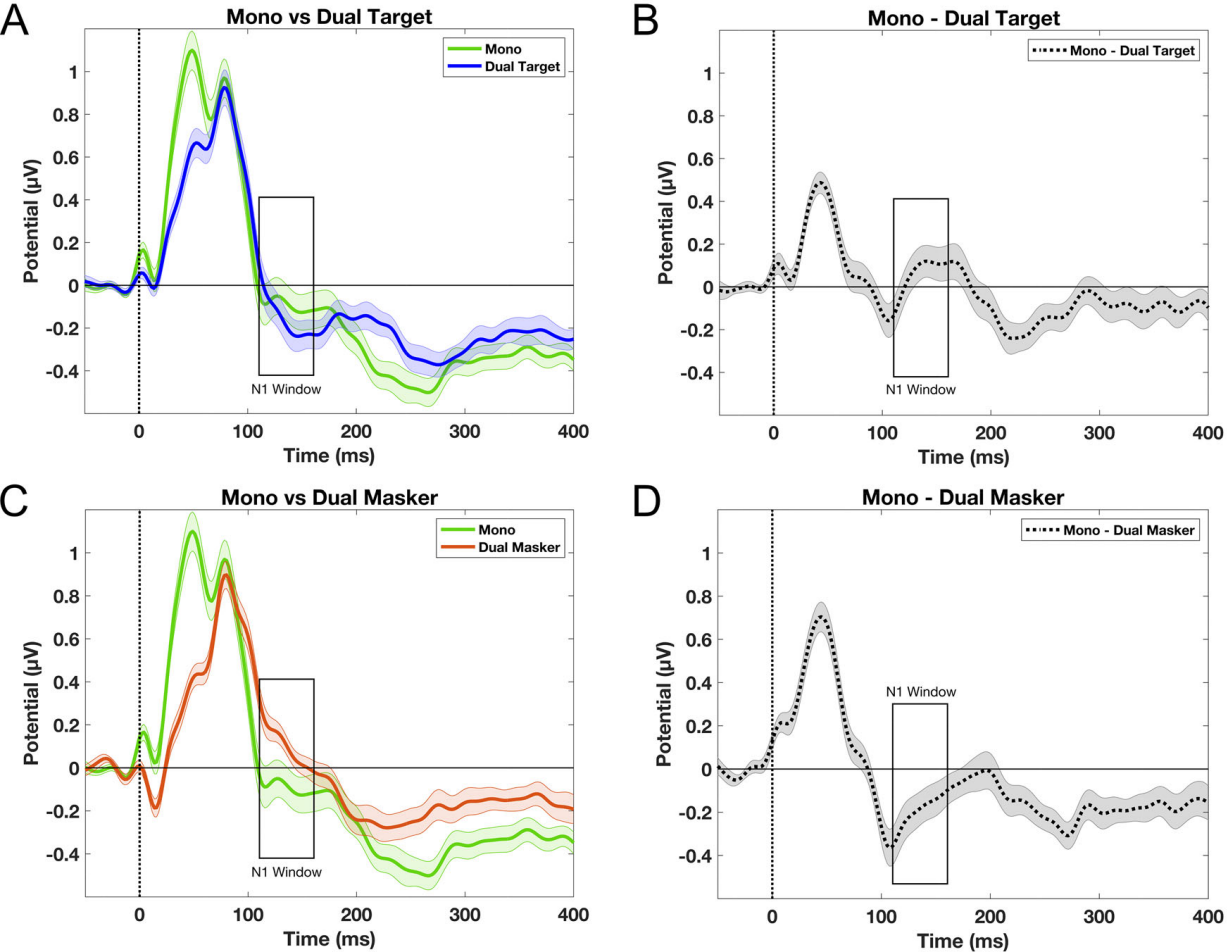
Attentional modulation of speech-evoked neural activity comparing mono- and dual-talker conditions at electrode Fz. Error bars indicate standard error. ***A***, Grand average ERP waveforms comparing the dual-talker target condition to the mono condition. ***B***, Difference waveforms (mono-target), where positive values indicate larger N1 amplitudes in the dual-talker target condition than in the mono condition. ***C***, Grand average ERP waveforms comparing the dual-talker masker condition to the mono condition. ***D***, Difference waveforms (mono-masker speech), where negative values indicate smaller N1 amplitudes in the dual-talker masker condition compared with the mono condition.

After analyzing the grand average waveforms for the target and masker story conditions, we compared them with a baseline mono condition, in which there was only one speaker for participants to attend to. This comparison was conducted to determine whether selective attention in a dual-talker scenario primarily boosts the neural response to the target speech, suppresses the response to competing speech, or involves both processes. The N1 amplitude did not differ significantly between the (dual) target and mono story conditions, as indicated by a Bonferroni-corrected paired *t* test (*t*_(23)_ = −0.790; *p* = 0.438; Fig. 4*A*,*B*). However, the N1 amplitude for the masker story condition was significantly smaller than that of the mono story condition (*t*_(23)_ = −2.798; *p* = 0.010), as shown in Figure 4, *C* and *D*.

### Predicting N1 amplitude differences

We used multiple regression analysis to assess whether performance on cognitive and psychoacoustic tasks could predict N1 amplitude differences between target and masker narratives (i.e., dual-talker condition only). As shown in Table 1, the multiple regression model demonstrated a strong fit.

**Table 1.** Results of the multiple regression analysis

	*β*	SE	*t*	*p*	GDW
Intercept	0.000	0.149	0.000	1.000	
Flanker	0.047	0.168	0.277	0.784	0.005
Stroop	0.598	0.178	3.370	0.003	0.166
TMT	−0.009	0.182	−0.518	0.611	0.026
Reading Span	0.649	0.167	3.894	0.001	0.278
TFS	−0.359	0.182	−1.971	0.064	0.108

Criterion variable: N1 amplitude difference between target and masker stories in the continuous multitalker spatial attention task. Predictors: performance on (1) Flanker, (2) Stroop, (3) TMT, (4) TFS, and (5) Reading Span. All variables were *z*-standardized. GDW indicates the relative importance of each predictor in the model. Overall adjusted *R*^2^ = 0.467; *p* = 0.005.

The N1 amplitude difference between target and masker narratives was significantly positively associated with reading span accuracy on three- and four-letter trials. This indicates that better working memory performance was associated with reduced differentiation in auditory cortical responses (i.e., closer to 0 or more positive N1 amplitude difference between target and masker). In contrast, the N1 amplitude difference demonstrated the opposite relationship with Stroop task reaction time differences between incongruent and congruent trials. Specifically, stronger behavioral inhibition abilities, in the form of reduced response interference (i.e., “smaller” Stroop RT difference values), were associated with more robust attentional effects in the auditory cortex (i.e., larger target- compared with masker-evoked N1 responses).

**Figure 5.**
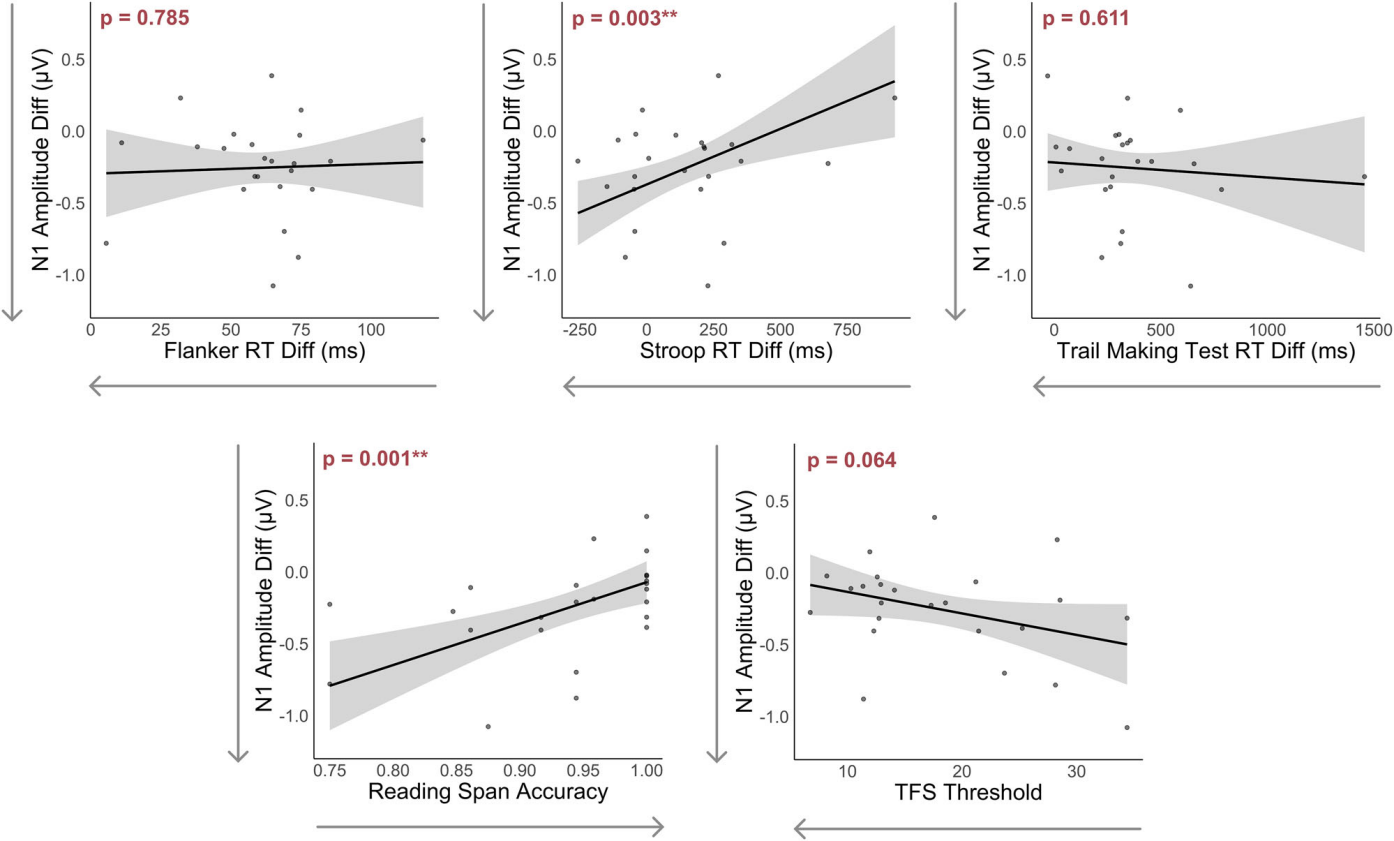
Effect plots illustrating the relationship between each predictor and the N1 amplitude difference. All other predictors are held constant. The shaded regions represent 95% confidence intervals for the N1 amplitude differences. N1 amplitude differences are calculated as the N1 amplitude for the target talker minus that of the masker talker; lower values on the *y*-axis correspond to larger differences in N1 amplitude between the target and masker streams. Arrows on the *x*-axis denote better performance on the cognitive and psychoacoustic tests.

**Figure 6.**
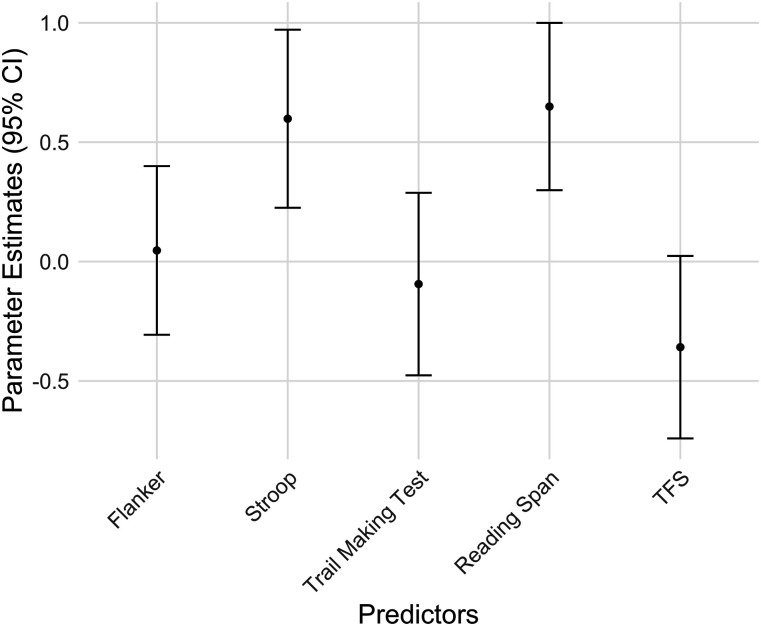
The 95% confidence intervals for the regression coefficients of each predictor.

TFS threshold and reaction time differences between incongruent and congruent trials on the Flanker and TMT were not statistically significant predictors. However, there was a trend suggesting that better TFS ability (indicated by lower thresholds) was associated with smaller N1 amplitude differences. Figure 5 illustrates the relationships between each individual predictor and N1 difference amplitudes. Additionally, Figure 6 provides further insights by depicting the confidence intervals for each predictor's coefficients.

**Figure 7.**
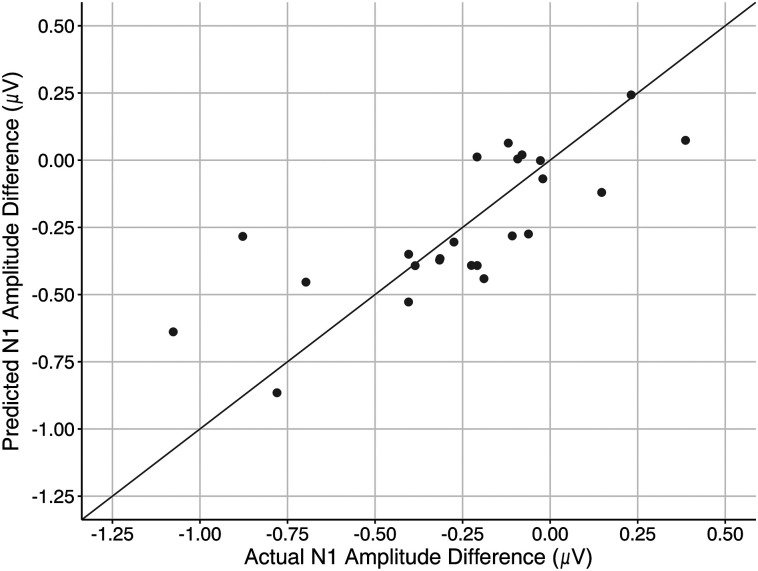
Cognitive and psychoacoustic abilities predict attentional modulation of speech representations. Results of the LOOCV. The graph shows the predicted and actual N1 amplitude differences for each participant.

LOOCV was used to validate the model fit and assess its predictive performance. The scatterplot in Figure 7 illustrates the relationship between the actual N1 amplitude differences (*x*-axis) and the predicted N1 amplitude differences (*y*-axis) generated from the LOOCV procedure. Each point on the plot represents an individual observation used as the validation set in a single iteration of LOOCV. The diagonal line indicates perfect prediction where predicted values match the actual values. Points closely aligned with this line indicate accurate predictions, while points further away indicate prediction errors. The LOOCV analysis resulted in a RMSE of 0.215 µV, reflecting the average magnitude of prediction errors for the N1 amplitude differences.

As previously mentioned, none of the six predictors exhibited strong collinearity, as indicated by low VIFs, suggesting that the model and its parameter variances should be stable. Nonetheless, the predictors were mildly correlated (Table 2). In such situations, relying solely on regression coefficients to assess predictor importance can be misleading. To address this, we employed the “dominance analysis” method (Budescu, 1993) to evaluate the relative importance of each predictor in the regression model. The results revealed that Reading Span had the highest GDW, followed by Stroop, indicating that working memory and response inhibition play significant roles in driving neural encoding differences between target and masker speech (Table 1). Notably, these findings align with the results of the multiple regression analysis, where Reading Span emerged as the most significant predictor, followed by Stroop. Additionally, TFS showed a relatively high GDW, consistent with its marginal significance (*p* = 0.07) in the regression model.

**Table 2.** Pairwise Pearson's correlation coefficients of the performance on cognitive tasks

	Flanker	Stroop	TMT	Reading span	TFS
Flanker	*r* = 1.000	*r* = −0.369	*r* = 0.065	*r* = 0.245	*r* = −0.127
		*p* = 0.076	*p* = 0.763	*p* = 0.248	*p* = 0.555
Stroop		*r* = 1.000	*r* = 0.156	*r* = −0.393	*r* = 0.196
			*p* = 0.467	*p* = 0.057	*p* = 0.358
TMT			*r* = 1.000	*r* = −0.023	*r* = 0.520
				*p* = 0.917	*p* = 0.009
Reading Span				*r* = 1.000	*r* = −0.075
					*p* = 0.727
TFS					*r* = 1.000

Reliability measures were computed for each predictor variable. Cronbach's alpha values were 0.88 for the Flanker task, 0.67 for the Stroop task, and 0.75 for the Trail Making task. For the Reading Span task, KR20 was 0.67. Due to the nature of the adaptive procedure, TFS thresholds were not assessed for reliability. The reliability of the N1 amplitude difference was assessed using the intraclass correlation coefficient (ICC, 0.353), calculated based on two-target and two-masker stories per participant.

**Figure 8.**
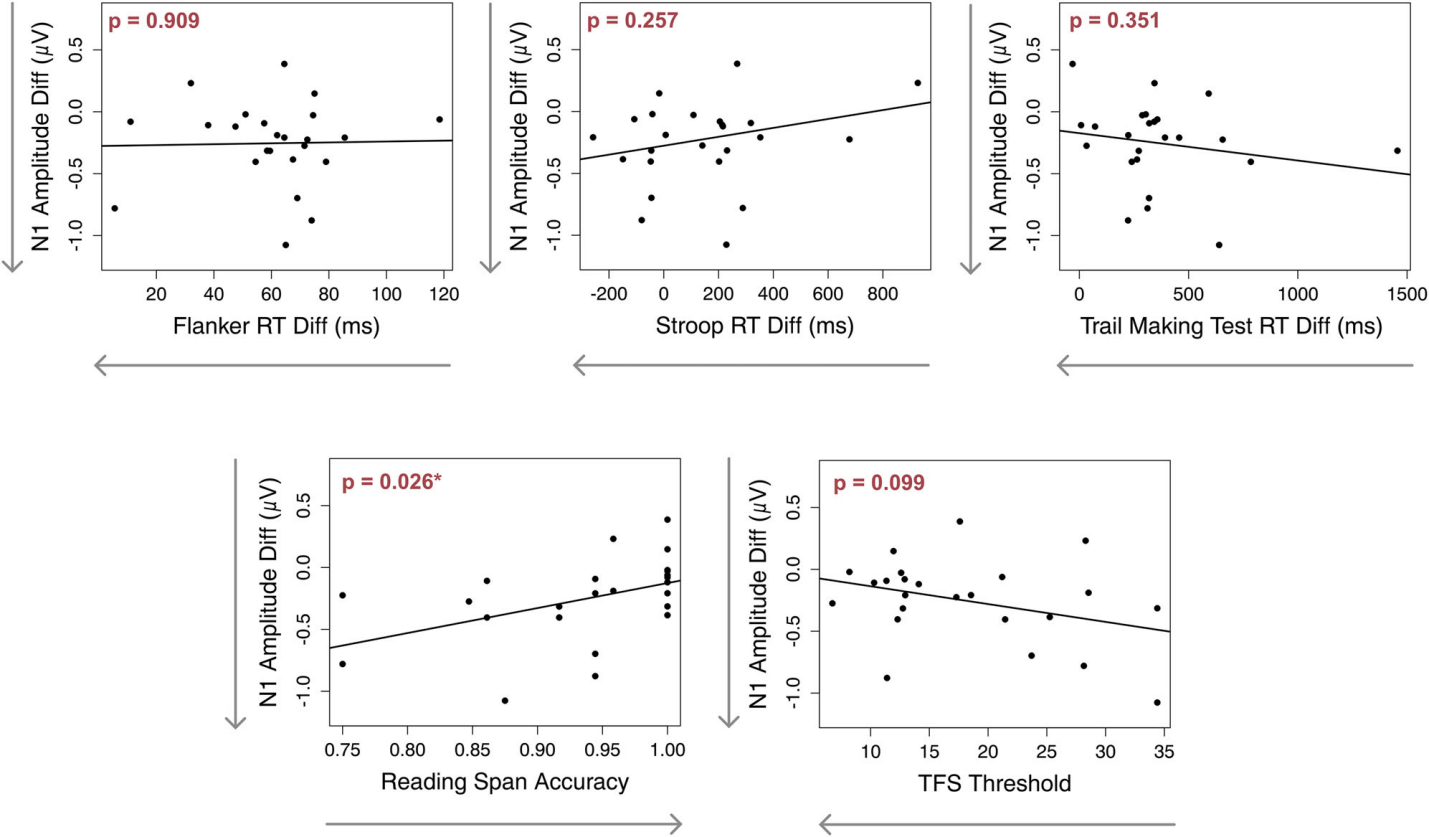
Bivariate correlations between each predictor and the N1 amplitude differences. Arrows on the *x*-axis denote better performance on the cognitive and psychoacoustic tests.

Finally, we also conducted bivariate correlations to examine the relationships between each predictor and N1 amplitude differences. The correlation plots summarizing these relationships are presented in Figure 8.

### Predicting behavioral performance of speech perception in noise

**Figure 9.**
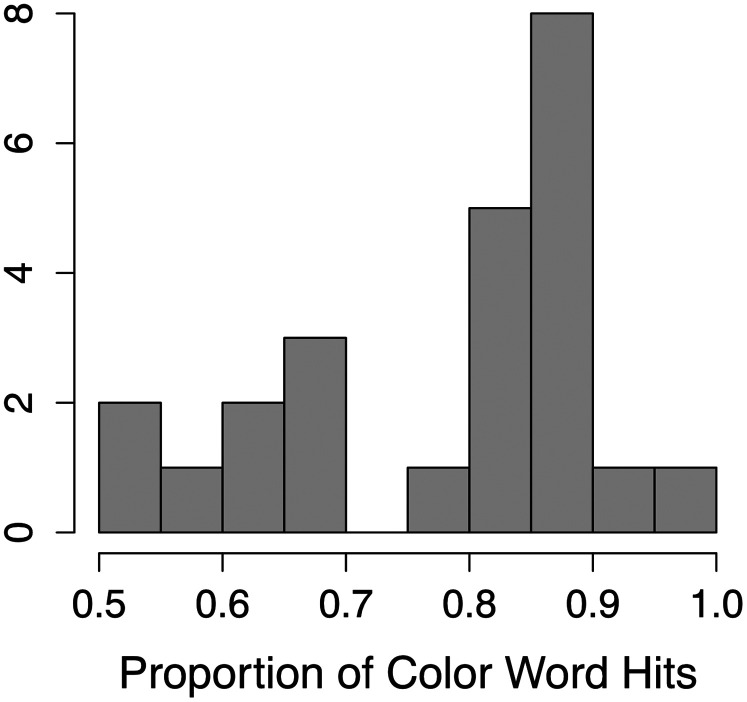
Histogram illustrating the distribution of proportion of color word hits in the continuous multitalker spatial attention task. Arrows on the *x*-axis denote better performance on the cognitive and psychoacoustic tests.

**Figure 10.**
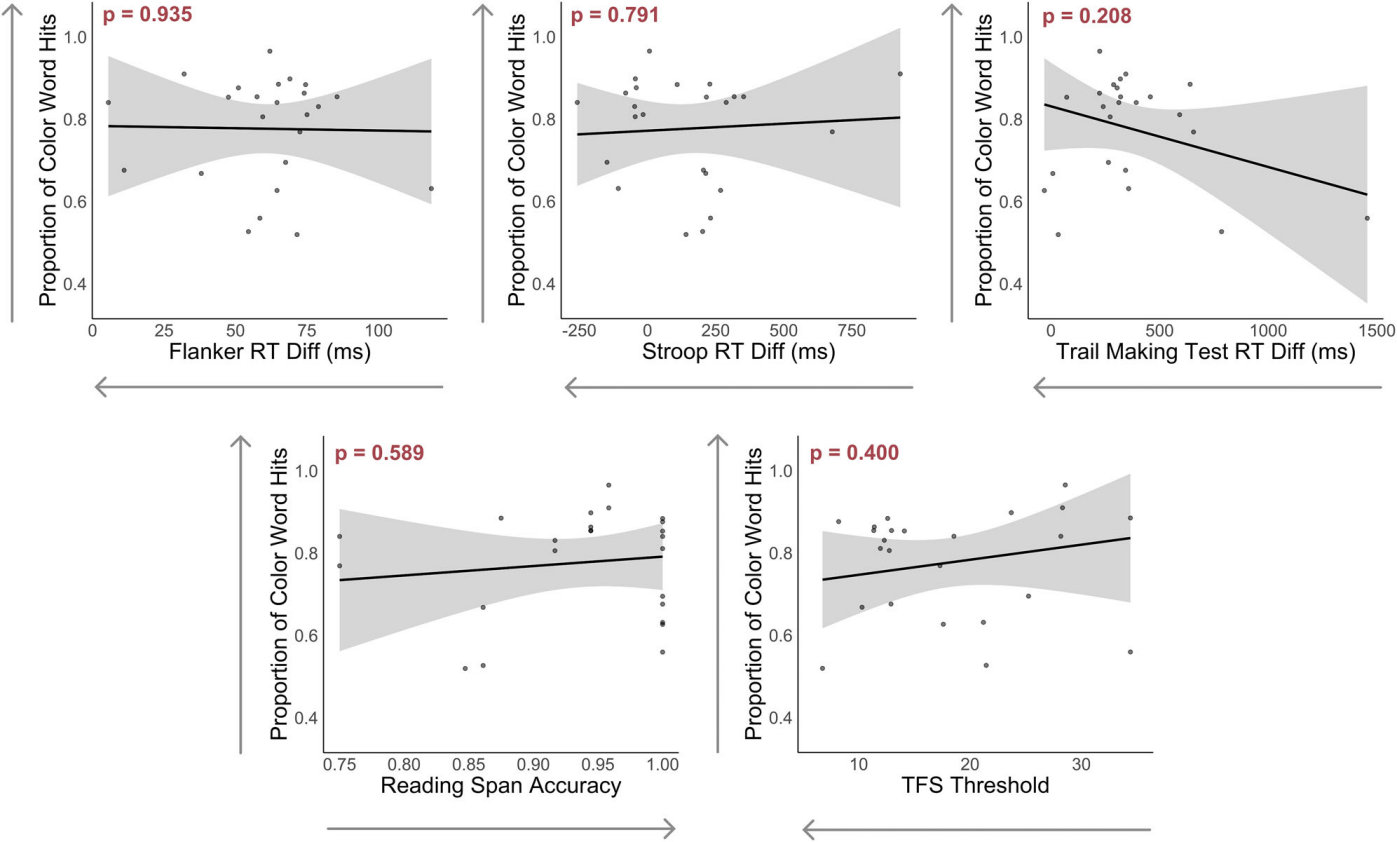
Effect plots illustrating the relationship between each predictor and the proportion of color word hits. All other predictors are held constant. The shaded regions represent 95% confidence intervals for the proportion of color word hits.

The earlier models explored the relationships between neural responses and performance metrics obtained separately from the EEG session. However, during the EEG session, participants also engaged in behavioral tasks that approximate real-world listening abilities. To further explore these relationships, we conducted an additional analysis using the same predictors to predict the proportion of color word hits, defined as the proportion of correctly identified color words in the target story within 2 s of their onset (i.e., excluding overlapping color words across the story pairs; Fig. 9). This measure was selected because it directly reflects the participants’ ability to focus on the target speech and respond promptly, making it a relevant and sensitive measure of real-world listening performance. We aimed to assess how well the observed relationships of the predictors with neural activity translate to behavioral outcomes. Based on this multiple regression model, none of the predictors were correlated with the proportion of color word hits (Table 3). This finding suggests that while cognitive and psychoacoustic abilities can accurately predict neural activity during SiN perception, they do not appear to predict SiN behavioral performance in this cohort. The relationship between each predictor and the proportion of color word hits is illustrated in Figure 10.

**Table 3.** Results of the second multiple regression analysis

	*β*	SE	*t*	*p*	GDW
Intercept	0.000	0.218	0.000	1.000	0.000
Flanker	−0.020	0.246	−0.082	0.935	0.003
Stroop	0.070	0.260	0.269	0.791	0.002
TMT	−0.348	0.267	−1.305	0.208	0.067
Reading Span	0.135	0.244	0.551	0.589	0.009
TFS	0.230	0.267	0.861	0.400	0.020

This model is using the same five cognitive and psychoacoustic predictors to predict the proportion of color word hits in the continuous multitalker spatial attention task. All variables were *z*-standardized. Overall adjusted *R*^2^ = −0.144; *p* = 0.828.

### Relationship between N1 amplitude differences and proportion of color word hits

**Figure 11.**
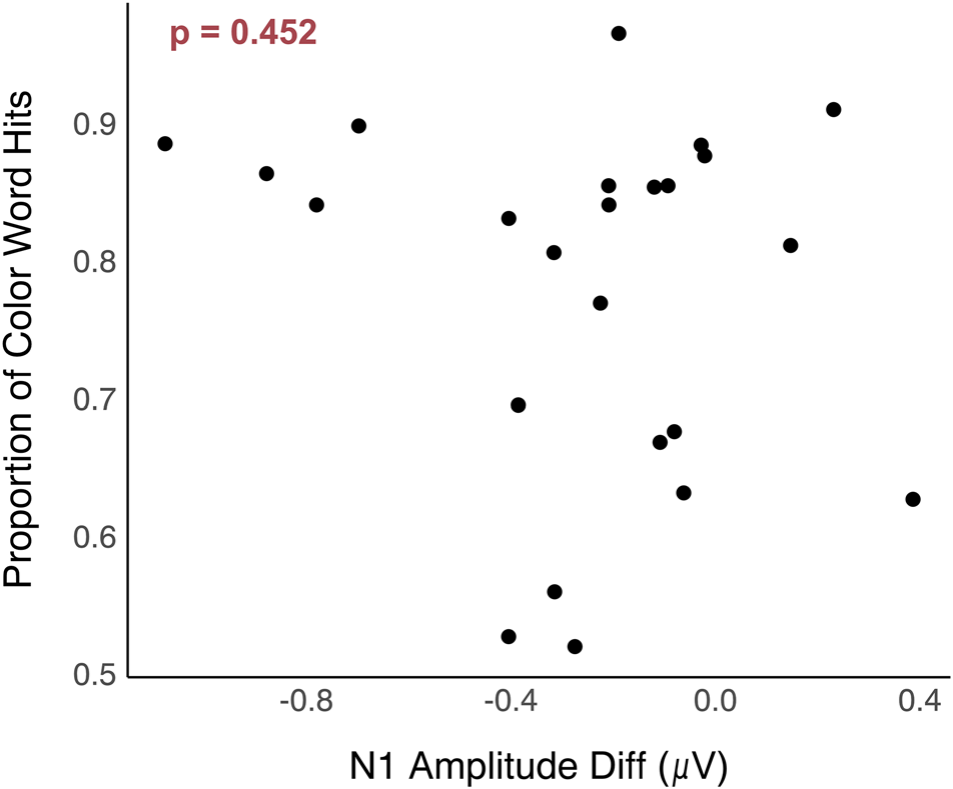
A scatterplot showing the relationship between the proportion of color word hits and N1 amplitude differences. Arrows on the *x*-axis denote better performance on the cognitive and psychoacoustic tests.

To gain further insight into the relationship between neural processing and speech perception, we performed a simple linear regression between the N1 amplitude differences and the proportion of color words that participants correctly identified. N1 amplitude differences between target and masker speech were not significantly associated with this ecologically valid SiN performance metric (Fig. 11).

## Discussion

In this study, we aimed to identify the key predictors of neural processing within the auditory cortex (specifically N1 amplitudes) associated with differentiating target from masker speech in a multitalker spatial attention task among normal-hearing listeners. For clarity, we adhered to well established interpretations of each cognitive predictor, emphasizing the constructs most relevant to our dynamic SiN EEG task. Specifically, Reading Span gauged working memory abilities, the Stroop task measured linguistic interference and inhibitory control, the Flanker task assessed selective attention and stimulus inhibition, and the TMT evaluated broader executive functions, including cognitive–attentional switching and processing speed. Additionally, TFS, a psychoacoustic measure, was included as it reflects sound localization abilities.

### Predicting N1 amplitudes

Our multiple regression model, which included five cognitive and nonaudiometric psychophysical predictors, demonstrated that working memory performance and inhibitory control significantly contributed to the variance in N1 amplitude differences between target and masker talkers. TFS also showed a trend toward further explaining additional variance. These findings demonstrate that, even among listeners with normal hearing, neural encoding of auditory selective attention during SiN listening varies based on domain-general abilities. This highlights the complexity of neural mechanisms involved in auditory selective attention during SiN, which span from basic auditory processing to higher-level cognitive functions.

Our multivariate approach further examines the relationship between each predictor and N1 amplitude differences between target and masker stories. In our study, better performance on the Stroop task—indicated by smaller reaction time differences between incongruent and congruent conditions—was associated with larger N1 amplitude differences between target and masker stories. This finding aligns with our hypothesis that better cognitive abilities, such as response inhibition, lead to larger N1 amplitude differences due to more effective suppression of distractor information and improved attention to target speech. The Stroop task is designed to assess linguistic interference and inhibitory control (Stroop, 1935; Troyer et al., 2006). This task requires participants to inhibit the automatic tendency to read the word and instead name the ink color, demonstrating their ability to suppress irrelevant information. This skill is likely transferable to complex listening environments, where participants must focus on target speech while inhibiting masker speech. Individuals with stronger inhibitory control may be better at suppressing neural representations of distracting information, allowing for enhanced focus on the target speech.

To test whether the N1 amplitude difference was driven by target enhancement or masker suppression, we compared the average N1 amplitudes of the dual-talker conditions to the single-talker (mono) condition within the continuous multitalker spatial attention task. The average N1 amplitude for the masker stream was smaller than the single-talker condition. In the single-talker condition, there is no need for inhibitory control as there are no competing stimuli. However, in a dual-talker scenario, effective inhibition of irrelevant speech is crucial. Thus, these results indicate individuals with better inhibitory control have significantly inhibited N1 responses to masker streams. This supports previous findings showing that the N1 is sensitive to attentional processes (Luck, 1995), with larger N1 amplitude differences reflecting greater attentional resource allocation for the target speech (Picton and Hillyard, 1974; Stapells, 2009; Kerlin et al., 2010).

Notably, accuracy on the Reading Span task for three- and four-letter trials was a significant predictor, emphasizing the role of working memory in neural encoding processes during challenging listening scenarios. Interestingly, higher accuracy on the Reading Span task was associated with *smaller* N1 amplitude differences, contrary to our hypothesis. Individuals with stronger working memory abilities are likely better at holding and manipulating information about the target speech. As a result, they may be more capable of using this information to predict upcoming auditory input, which is crucial for understanding speech, particularly in noisy environments (Akeroyd, 2008; Obleser and Eisner, 2009). These predictive processes might reduce the need for effortful processing of target speech, as the brain can rely on expectations for comprehension. Conversely, individuals with poorer working memory may struggle to predict and preprocess information in the target stream, therefore requiring a greater reliance on real-time processing. This may increase cognitive load and demand more neural resources for target speech focus and distractor suppression, potentially explaining larger N1 amplitude differences in these individuals.

Interestingly, in our pilot testing, the total Reading Span accuracy of all trials (3–7 sentence–letter pairs) was not a strong predictor of N1 amplitude differences between target and masker speech. This finding suggests that accuracy on three- and four-letter trials may be a more informative measure of working memory capacity related to neural processes in SiN perception. One possible explanation is that tasks involving fewer items (three to four letters) fall within the working memory capacity limit (Cowan, 2001, 2015). This smaller capacity measure might be more sensitive to individual differences in neural efficiency and attentional control during auditory attention tasks. When the Reading Span exceeds this capacity (five to seven letters), performance may be influenced by additional factors such as strategies, chunking, or long-term memory processes, which may not directly reflect the working memory capacity relevant to real-time speech processing in noise. Consequently, focusing on moderate working memory capacities may provide a more accurate representation of the cognitive resources used during complex listening tasks, leading to better predictions of neural activity differences in multitalker auditory environments.

TFS thresholds did not significantly predict N1 amplitude differences. Nevertheless, as shown in Figure 5, there is a notable trend suggesting that better TFS thresholds are associated with smaller N1 amplitude differences between target and masker speech. This trend contradicts our initial hypothesis, where we expected better TFS sensitivity to correspond to larger N1 amplitude differences. TFS processing begins in the peripheral auditory system, starting with basilar membrane filters in the cochlea and extends through the brainstem (Plomp, 1964; Borjigin, 2023). These processes are important for encoding interaural timing differences, which are crucial for separating target and masker speech based on azimuthal location. In SiN perception, this suggests that TFS processing contributes to the early spatial segregation of auditory streams, likely occurring at the cochlea and brainstem levels. Individuals with better TFS sensitivity may exhibit larger neural differentiation of speech streams in these early stages of auditory processing. This enhanced peripheral processing may reduce the need for differentiation or attentional modulation in the cortex, as the target and masker streams may already be separated when they reach the auditory cortex. This may explain why better TFS sensitivity is associated with smaller N1 amplitude differences, reflecting less cortical effort for stream segregation. TFS was tested at a fundamental frequency (F0) that differs from the F0 of the male talkers used in this study. Specifically, the TFS F0 was selected to be approximately halfway between the male and female (resynthesized) voices identified during pilot recordings. While this choice allowed us to test TFS performance in a frequency range relevant to both genders, it introduces a potential limitation in directly relating TFS thresholds to the cortical processing of male voices. This highlights the need for further research to better understand the relationship between peripheral auditory processing and cortical neural responses, particularly in challenging listening scenarios.

The TMT reaction times were not significant predictors of N1 amplitude differences in our study. The TMT primarily assesses executive functioning in a visuomotor domain by requiring participants to switch attention between numerical and alphabetical sequences (e.g., connecting 1 to A, 2 to B) in an ascending order. While our multitalker spatial attention task also involves attentional switching, the type of switching required in the TMT may not align with the demands of auditory attention in SiN perception. The attentional switching in our continuous multitalker task involves dynamically redirecting attention between left and right ears as the target story alternates between spatial locations throughout its narration. This process requires not only attentional control but also the integration of spatial auditory cues to track the target stream while suppressing competing distractor speech. These demands are likely supported by domain-specific auditory and linguistic mechanisms, which involve distinct neural pathways optimized for processing spatial and linguistic auditory information. This distinction highlights the importance of task-specific predictors when studying complex auditory tasks. Further research could explore whether attention-switching measures within the auditory domain, or involving linguistic components, provide stronger associations with neural indices like N1 amplitude differences.

Flanker test reaction times were not significant predictors of N1 amplitude differences in our study. Although the Flanker test is a well established measure of selective attention in (visual) space, it may not fully capture the neural processes required to differentiate between target and masker speech in a noisy environment. The Flanker, like the Stroop, also assesses inhibitory control, but does so in a different context. The Stroop test requires participants to inhibit a more automatic linguistic response (reading the word) in favor of a less automatic one (naming the ink color), directly engaging cognitive mechanisms related to speech and language processing. This linguistic interference is evidently relevant to the challenges of SiN perception, especially when maskers are meaningful speech rather than nonlinguistic sounds like steady-state noise, which does not trigger lexical activity (Lu et al., 2016; Schneider et al., 2022). In contrast, the Flanker test primarily involves visuospatial interference, where participants must inhibit responses to maskers visually adjacent to the target stimulus. This suggests that in individuals with normal hearing performing a realistic SiN task, linguistic interference is more impactful than (visuo-)spatial interference. Future work might determine whether this asymmetry also holds when listeners have hearing loss and, consequently, greater challenges with spatial masking.

### Implications for the N1

Our findings suggest a complex interaction between cognitive abilities and auditory processing, updating our understanding of the N1 component. Traditionally considered a marker of basic auditory processing and attention, the N1 also appears to be influenced by higher-order cognitive functions such as inhibition and working memory, particularly in challenging listening scenarios. Extensive research suggests that when individuals attend to specific auditory stimuli, the N1 amplitude is typically enhanced, indicating a greater neural response to the attended sounds (Hillyard et al., 1973; Picton and Hillyard, 1974; Stapells, 2009; Kerlin et al., 2010). Consistent with this, better response inhibition, as measured by the Stroop task, is associated with larger N1 amplitude differences. However, our findings from the Reading Span test indicate that this relationship is more complicated; higher working memory capacity is associated with “smaller” N1 amplitude differences between attended and unattended streams. This suggests that neural processing during speech perception is determined by compensations and compromises between top–down cognitive influences.

### Predicting behavioral performance of speech perception in noise

In addition to analyzing the direct relationship between cognitive performance and neural mechanisms of auditory selective attention (N1 amplitude differences), we also assessed how each cognitive predictor relates to ecologically relevant listening behaviors during the continuous multitalker spatial attention task, conducted simultaneously while EEG was recorded. Specifically, we tested the relationship of each cognitive predictor and the proportion of correctly identified color words in the target story. Interestingly, none of the cognitive and psychoacoustic predictors showed a significant correlation with this behavioral measure.

This lack of a significant correlation between cognitive abilities and SiN perception is consistent with previous findings in normal-hearing individuals. Füllgrabe and Rosen (2016) found that working memory capacity is associated with SiN intelligibility in older, hearing-impaired individuals, but this correlation is absent in younger, normal-hearing listeners. One possible explanation is that age-related auditory deficits force older, hearing-impaired individuals to rely more heavily on working memory-based compensatory mechanisms for successful SiN perception. In contrast, younger listeners may depend less on cognitive resources due to better auditory processing. However, other studies, such as Oberfeld and Klöckner-Nowotny (2016), have demonstrated significant associations between cognition and SiN perception. The meta-analysis by Dryden et al. (2017) highlights three key factors that could explain these mixed findings: differences in cognitive measures, variability in SiN tasks, and participants’ hearing thresholds. Future research should consider these factors to better understand the nuanced role of cognition in SiN processing.

Additionally, N1 amplitude differences were not associated with SiN perception (proportion of color word hits) in our study. The N1 component has been widely associated with SiN perception in previous studies (Parbery-Clark et al., 2011; Billings et al., 2013; Bidelman and Howell, 2016). The lack of association between N1 amplitudes and behavioral SiN performance in our study may be due to differences in the tasks used to assess SiN perception. Conventional measures such as the hearing-in-noise test (Parbery-Clark et al., 2011), QuickSiN (Bidelman and Howell, 2016), and sentence-in-noise identification tasks (Billings et al., 2013) assess word or sentence identification in noise. These tasks require participants to process and repeat semantically meaningful speech. In contrast, our SiN task required participants to detect and respond to embedded color words in a continuous multitalker spatial attention paradigm, measuring their ability to selectively attend to the target stream and suppress distractors. This task emphasized rapid attentional shifts and target word detection, rather than the linguistic reconstruction and sentence-level recognition typically evaluated in more conventional SiN measures. Task differences may explain the lack of correlation between N1 amplitudes and SiN perception, emphasizing the need to consider experimental design when interpreting neural–behavioral relationships. Future studies should investigate how task demands and behavioral measures assess distinct facets of SiN processing.

Although cognitive abilities and N1 amplitudes *were not* significantly associated with listening performance, it is noteworthy that cognitive abilities *were* significantly associated with N1 amplitudes. These findings highlight an important distinction between neural encoding of auditory selective attention and behavioral SiN performance. N1 amplitude differences reflect underlying neural mechanisms involved in focusing attention on target speech and suppressing masker speech, whereas behavioral SiN performance measured in our task likely involves additional factors and compensatory strategies. Given that SiN processing is multifaceted, no single behavioral speech test, such as color word detection, can readily parse the many underlying mechanisms that might contribute to it. In these cases, neural metrics can add substantially to our understanding which might not be fully captured by behavioral data alone.

Additionally, our cohort consisted of young, highly educated individuals, with all participants having completed at least 13 years of education. It is possible that the well educated status of these participants equips them with advanced compensatory mechanisms that may enable strong performance in speech perception tasks, despite differences in cognitive abilities. As shown in Figure 9, participants achieved a high proportion of color word hits, indicating strong overall performance. For those with lower cognitive task performance, their educational background and overall health may have provided them with effective strategies for maintaining high performance in challenging listening tasks. These strategies might include better use of contextual cues, more effective allocation of attentional resources, or superior problem-solving skills, allowing them to compensate for cognitive limitations. Consequently, the homogeneity in educational background might mask differences in cognitive performance, resulting in consistently strong performance in the continuous multitalker spatial attention task, regardless of cognitive predictor scores. Although our data do not allow definitive claims, the findings suggest that N1 amplitude differences might serve as early markers of auditory deficits or hearing loss, warranting further investigation in diverse populations.

### Limitations

A key limitation of this study is the relatively small sample size of 25 participants, which may limit the ability to fully capture the complexity and variability in individual differences, especially with multiple regression models involving five predictors. To improve the reliability and generalizability of these findings, future studies should prioritize larger samples to better represent the population and enhance the robustness of the results. Another limitation of this study is the scoring approach used for the working memory span task. We acknowledge that this method differs from the traditional scoring approach, which averages accuracy across all list lengths (Conway et al., 2005). Our choice was guided by theoretical accounts of working memory capacity (Cowan, 2001, 2015) and the need for a stable measure that reflected meaningful individual differences. Future research should explore how different scoring approaches impact findings to ensure robustness across methodologies. Additionally, in this study, the reliability of cognitive performance measures was adequate, with Cronbach's alpha and KR20 values ranging from 0.67 to 0.88. For the criterion measure, the N1 amplitude difference, the ICC was 0.353. This relatively low ICC may be partly attributable to the small sample size, which inherently increase variability and reduce the precision of reliability estimates. Future studies should aim to address this limitation by including a larger sample size to improve stability of reliability estimates.

## Conclusion

Overall, our findings highlight the complexity of the relationships between auditory processing, cognitive abilities, and attentional gains in speech perception under noisy conditions. We demonstrate that neural measures reflect impactful interindividual differences that are not fully captured by solely traditional assessments of audiometric hearing and behavioral performance. These findings have significant implications for improving hearing loss diagnostics, which currently rely primarily on ear health assessments. By incorporating cognitive abilities and neural activity measures, we can obtain a more comprehensive understanding of speech perception, even among normal-hearing listeners. Having established the predictive value of these tasks for neural processing of SiN, future work might further clarify how each of these reflects differences in underlying cognitive mechanisms across individuals. Understanding how the brain processes auditory information in noisy environments—and how differences in cognition influence this process—could lead to earlier detection of hearing loss, improved strategies for enhancing speech perception in daily life, and, ultimately, a significant improvement in the quality of life for individuals facing hearing challenges.

## Synthesis

Reviewing Editor: Anne Keitel, University of Dundee Decisions are customarily a result of the Reviewing Editor and the peer reviewers coming together and discussing their recommendations until a consensus is reached. When revisions are invited, a fact-based synthesis statement explaining their decision and outlining what is needed to prepare a revision will be listed below. The following reviewer(s) agreed to reveal their identity: NONE.

Thank you for providing a thorough revision and responses to the reviewers' concerns. One of the original reviewers (see below) and the editor assessed your revised manuscript and were happy with the changes. Congratulations on the acceptance of your manuscript!

Reviewer #1

Advances the Field (Required)

This research evaluates how cognition effects the auditory selective attention during speech perception. This concept is not well understood and this manuscript helps to further the field by identifying an approach that may allow for earlier detection of hearing problems.

Statistics

Reported the rational for a smaller sample size and including a limitation about it. The regression is supported.

Software Comments

A toolbox within MATLAB was used, not a new developer software.

Comments to the Authors (Required)

I appreciate the effort put in to revise this manuscript. I believe this revision is a substantial improvement from the original. I have no further comments that need addressed.
